# Magnetic properties of quenched binary and ternary quasicrystal approximants

**DOI:** 10.1038/s41598-024-81987-7

**Published:** 2024-12-17

**Authors:** Fernand Denoel, Takayuki Shiino, Yu-Chin Huang, Girma Hailu Gebresenbut, Cesar Pay Gómez, Roland Mathieu

**Affiliations:** 1 Department of Materials Science and Engineering, Uppsala University, Box 35, 751 03 Uppsala, Sweden; 2 Department of Chemistry, Ångström Laboratory, Structural Chemistry, Uppsala University, 751 21 Uppsala, Sweden

**Keywords:** Magnetic properties and materials, Structure of solids and liquids

## Abstract

The magnetic properties of binary Gd-Cd and ternary Gd-Au-Ge crystals obtained from the newly introduced low-melt peritectic formation (LMPF) synthesis method were investigated. This method consists of a rapid quenching of the metallic melt followed by an annealing treatment at a relevant temperature. In the first system, both quasicrystal (QC) and approximant crystal (AC) phases can be stabilized, whereas only the AC phase is obtainable in the pseudo-binary Gd-(Au-Ge) system. The magnetic properties of the crystals obtained from LMPF for each system are compared to those of larger single grain crystals obtained from the self-flux (SF) method.

## Introduction

**Fig. 1:**
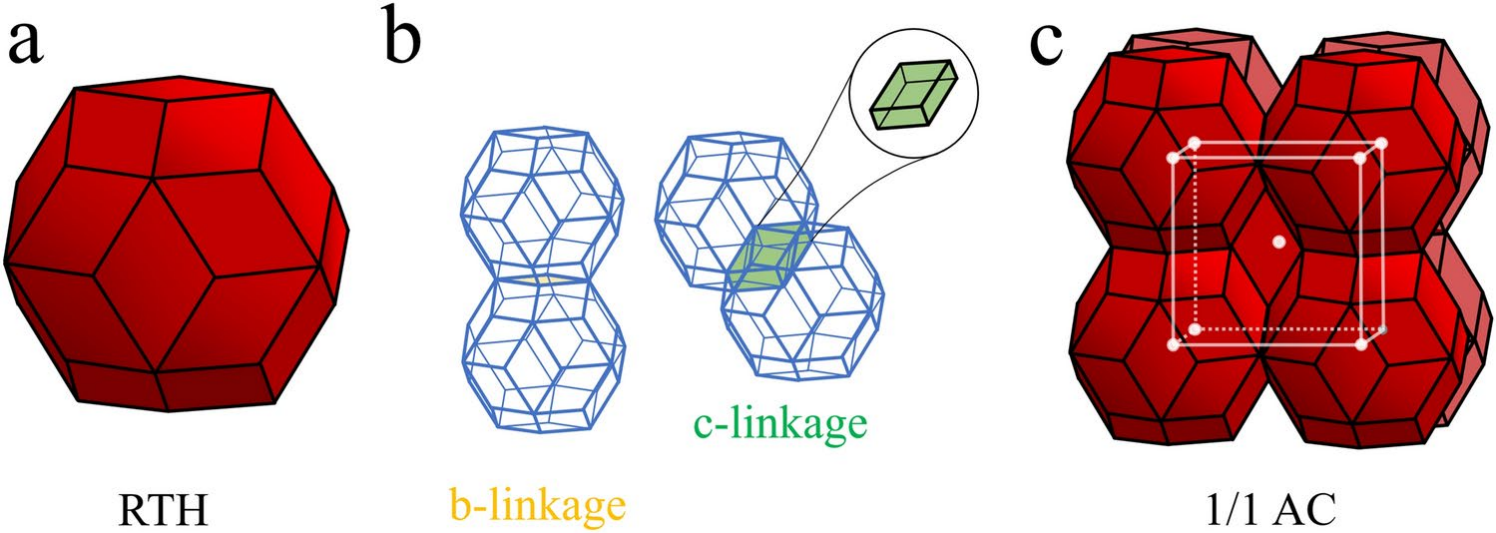
(**a**) The rhombic triacontahedron, the elementary volume used to describe Tsai QCs and ACs. (**b**) The two linkage rules applying to RTHs, either by sharing a face along a 2-fold axis (b-linkage) or by intersecting and sharing an obtuse golden rhombohedron along a 3-fold axis (c-linkage). (**c**) Representation of the unit cell of 1/1 ACs as a network of intersecting RTHs.

**Fig. 2:**
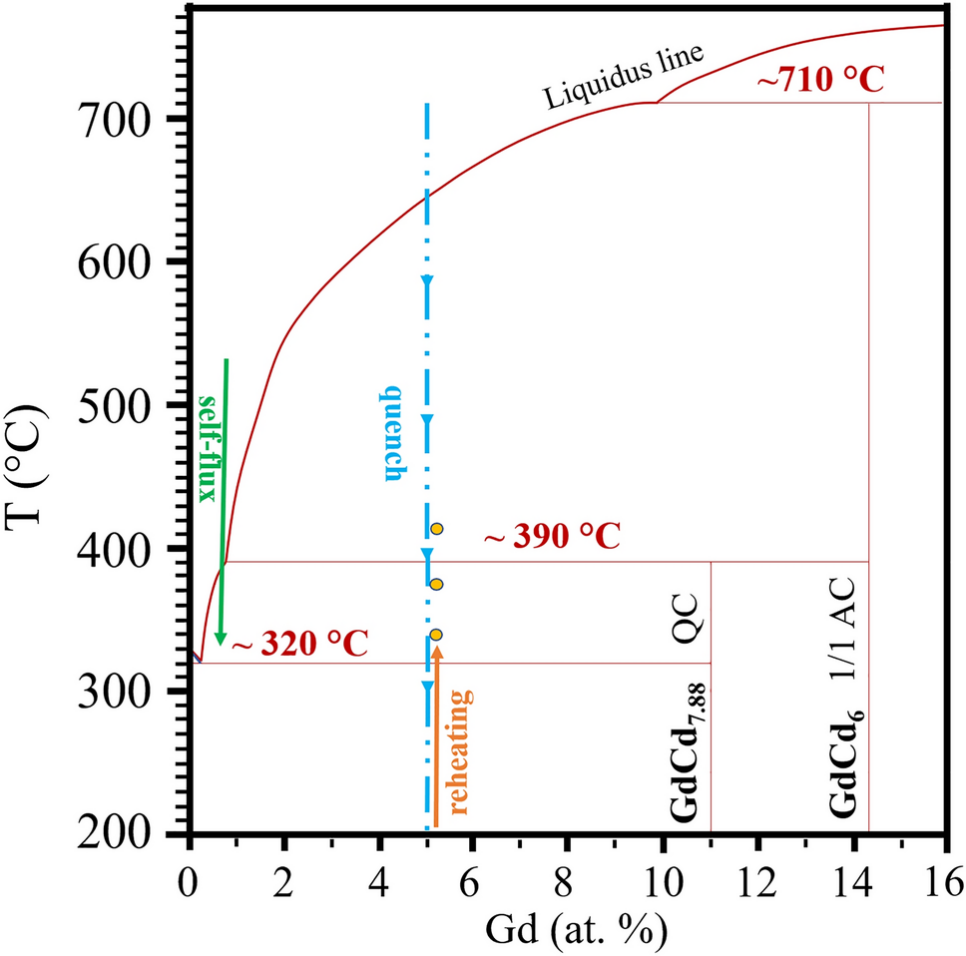
Binary Gd-Cd phase diagram. The self-flux (SF) is presented with a green arrow (slow cooling) whereas the low-melt peritectic method (LMPF) for synthesis is exemplified by the blue arrow (quench) and reheating (orange arrow) after which annealing is performed at the desired temperature *T*_*a*_ .

Quasicrystals (QCs) constitute an interesting state of matter with an aperiodic order along one or more dimensions^1^. Tsai-type QCs represent a class of QCs with icosahedral symmetry, which is aperiodic in all three dimensions. Approximant crystals (ACs) are a set of regular, periodic crystals, but with similar local environments compared to their QC counterparts. The most commonly studied type of Tsai QCs and ACs are binary, forming from a mixture of Cadmium and a choice of rare-earth^2,3^. The elementary building unit in both cases is the Tsai cluster, an atomic decoration of a rhombic triacontahedron (RTH), and consisting of concentric atomic shells all possessing icosahedral symmetry with the exception of the innermost (orientationally disordered) tetrahedron^4^. The most common type of AC are the 1/1 ACs, which possess a body-centered cubic packing of intersecting Tsai clusters, see Fig. 1. Another existing type of AC are the 2/1 ACs, with the naming convention originating from the cut-and-project approach used to obtain a quasiperiodic 3D crystal structure from a periodic hypercrystal defined in 6D. From this scheme, icosahedral quasicrystals can be obtained by “cutting” a 3D subspace from the 6D hyperspace with a slope of $\varphi$ = 1.618..., the golden ratio, with respect to the axes of the hypercrystal. The ACs are defined as the ratios of consecutive terms $F_n$ of the Fibonnacci sequence (0, 1, 1, 2, 3, 5, 8, 11, ...), the limit of which converges to $\varphi$. Cuts with rational parametrizations give periodic crystals, with the first two type of choices of slope matching the geometries of real crystals: the 1/1 and 2/1 ACs^5^. The self-flux (SF) method allows one to grow large size, high quality crystals from a slow cooling procedure, although Tsai-type QCs can only form at very small rare earth concentration in the starting materials, typically for R_y_Cd_100-y_ with *y* < 1 (R : Gd,..., Lu)^2^. Instead of the SF method slow cooling allowing to select the phase, the low-melt peritectic formation (LMPF) synthesis method^6^ can be used for starting materials with *y* values too large to obtain QCs using the self-flux method, but select this phase from the annealing temperature to increase the yield to that of 1/1 ACs, typically at *y* > 5. The quenching of the starting materials for the LMPF method can be performed by example by quickly dipping the sealed stainless steel ampule used for synthesis in liquid nitrogen to immediately solidify it. In the case of the binary Gd-Cd system, the material is then reheated to a temperature $T_a$ where Cd is in its liquid form but the QC and/or AC phases are stable, and left to anneal there for some time. Depending on the choice of $T_a$, a mixture of the two phases, QC and 1/1 AC, are present with different ratio, see Fig. 2. For a choice of low enough $T_a$, this synthesis method can be used to obtain large quantities of the QC phase with similar physical and magnetic properties, as we will observe later.

A peritectic reaction is a reaction where a solid phase $\alpha$ surrounded by liquid and kept at a specific temperature transforms into a second phase $\beta$. In the case where *y* = 5, the predominant phase present for no —or short— annealing ($\lesssim$ 2 hours) is 1/1 AC, as evidenced from powder X-ray diffraction (PXRD) measurements^6^. The phase $\alpha$ is the 1/1 AC, but by annealing the quenched sample at the right temperature, the quasicrystalline phase $\beta$ can be obtained. Previous investigations of the LMPF method in the Gd-Cd, Gd-Y-Cd, Gd-Cd-Mg and Gd-Cd-Zn systems have been used to establish how the phase diagrams and peritectic decomposition temperatures were altered compared to Gd-Cd QCs and ACs obtained from SF method. In the present work, we investigate how the magnetic properties of crystals synthesized using the two methods change in the Gd-Cd and Gd-Au-Ge systems. In the case of the pseudo-binary Gd-(Au, Ge) phase diagram, unlike for the Gd-Cd one, no stable QC phase exists^7^. Ternary Gd-Au-Ge samples synthesized in this investigation are thus all 1/1 ACs, for both the SF and LMPF method. Also, we note that chemical disorder is always present in the GdCd_7.88_ QC^2,8^ and the Gd-Au-Ge 1/1 AC phases^9^, while the GdCd_6_ 1/1 AC exhibits perfect chemical order^3,10^. Whereas the binary R-Cd AC system shows long-ranged order antiferromagnetic in nature^3,11–13^, ternary systems R-(Au, M) exhibit a wider range of magnetic behaviors, including spin glass transition, ferro- and ferrimagnetic states, as well as antiferromagnetic states^14–16^. This diverse set of magnetic behaviors may emerge from the interplay of local distortions due to chemical mixing, the RKKY interaction depending on nearest magnetic ion distances, and the local electron concentration for compounds with different chemical compositions^17^. Using the LMPF method along with differential scanning calorimetry (DSC) allows for a much faster investigation of new phase diagrams, and is thus of particular importance to quickly obtain map pseudo-binary phase diagrams, for example in R-(Au, M) with M = Si, Al, Ge, Ga.

The choice of M = Al allows for the widest range of composition, and therefore a wide range of average number of valence electron per element (the *e/a* ratio), a metric widely used as an empirical criterion that has been correlated to the type of magnetic ordering in a diverse set of QCs and ACs^16^. In addition, for M = Al, the conventional atomic structure of the Tsai cluster building block is always preserved, with an orientationally disordered tetrahedron at its center, whereas other choices of the element M can lead the tetrahedron to be partially or entirely replaced by a rare-earth atom (pseudo-Tsai cluster)^18^. By choosing M = Si, it is possible to tune ternary 1/1 ACs to have either entirely the inner tetrahedron (regular Tsai type) or a rare earth at the cluster center (pseudo-Tsai type) based on the synthesis conditions and compare the magnetic properties of each independently^18,19^. This choice is more limiting in terms of the range of Au concentrations, but allows a complete control of atomic-scale tuning at the cluster center. This substitution at the Tsai cluster center can affect dramatically the static and dynamic magnetic properties of the samples concerned, due to the center rare-earth atom increasing the magnetic frustration and altering the magnetic structure^19,20^.

In this work we have investigated LMPF-grown Gd-based QCs and 1/1 ACs of binary Gd-Cd and ternary Gd-Au-Ge systems. We report the first synthesis of ternary Au-based ACs using the LMPF method, although other methods for quenching methods have been used, e.g., melt-spinning^21,22^. It is rather unclear whether samples obtained using different quenching method would have identical properties. The magnetic properties of all aforementioned samples have been measured and compared to the single-grain self-flux samples of the same phase, and the results discussed in the light of their structural and morphological differences.

## Results and discussion

The peritectic formation, illustrated in Fig 2 consists of a reaction between the liquid melt and the micro-crystallites, allowing to select a region of the phase diagram that results in less flux and more crystal samples compared to the self-flux method. In the following, we will refer to samples synthesized with different identifiers depending on the synthesis method used: SF for the self-flux method and LMPF for the low-melt peritectic formation method accompanied by the final annealing temperature $T_a$ (relevant only for LMPF samples). The annealing duration was set to 100 hours in all samples.

**Fig. 3:**
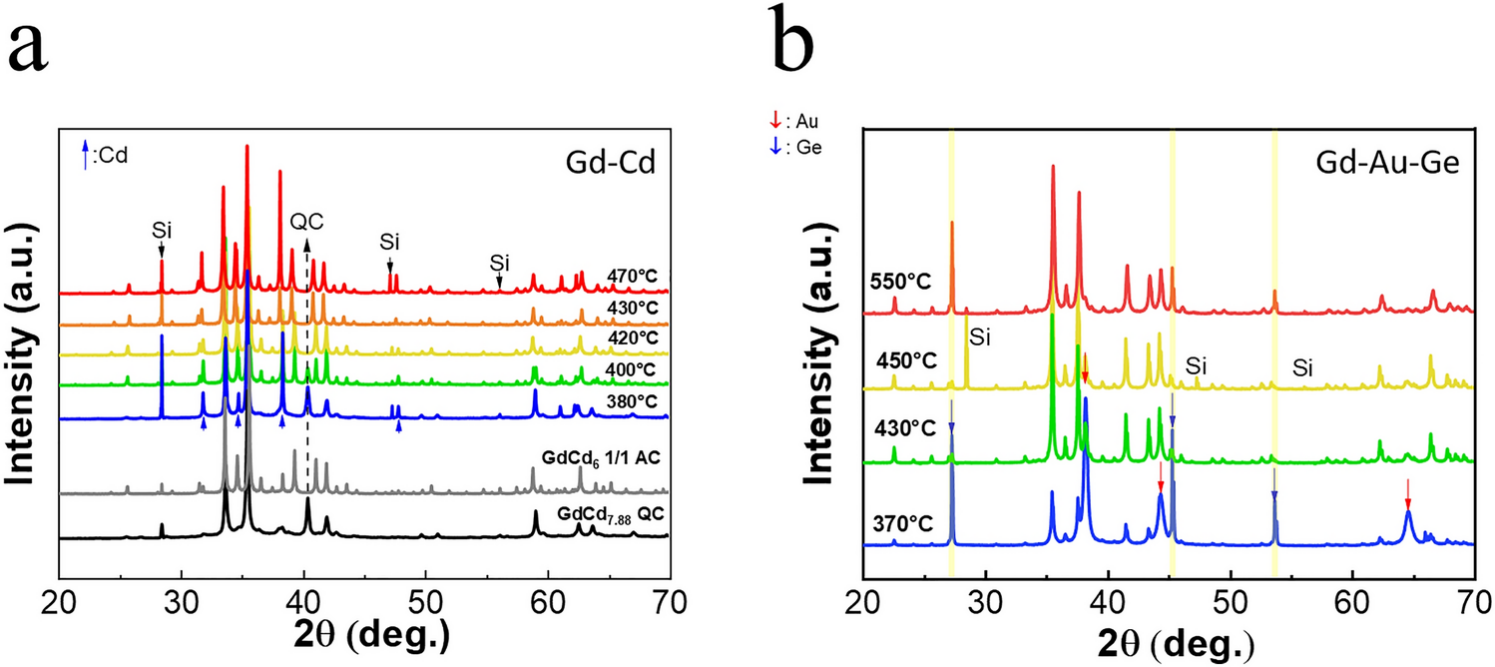
PXRD patterns of LMPF-grown (**a**) Gd-Cd and (**b**) Gd-Au-Ge for various annealing temperatures $T_a$. In panel (**a**), the PXRD patterns of SF-grown GdCd_6_ 1/1 AC and GdCd_7.88_ QC crystals are added for reference. The black dashed line highlights a characteristic reflection of the QCs in the binary Gd-Cd LMPF samples (see also Fig. S2).

The PXRD patterns shown in Fig. 3 are similar to those of QCs and ACs obtained using LMPF synthesis. The extra Si was added as a calibration reference before acquiring the PXRD patterns of all samples. We observe that in the Gd-Cd system, the LMPF-grown crystals obtained with $T_a$ > 380 $^{\circ }\hbox {C}$ display a similar diffraction pattern as SF-grown ACs. All of these crystals were determined to be 1/1 ACs. In the case of the crystal grown at $T_a$ = 400 $^{\circ }\hbox {C}$, an admixted QC phase was also observed in the diffraction data. On the other hand, the LMPF-grown crystal obtained at the lowest temperature ($T_a$ = 380 $^{\circ }\hbox {C}$) displays an PXRD pattern typical of QCs, with additional reflections from the Cd flux; note e.g. the reflection marked by a dotted line in Fig. 3a in the patterns of the low-temperature annealed LMPF-grown Gd-Cd samples. For clarity, the PXRD patterns of the SF-grown binary GdCd_7.88_ QC and GdCd_6_ 1/1 AC are shown in Fig. S2. The PXRD data of the LMPF 1/1 ACs, supplemented by room temperature atomic structure refinement from single crystal X-ray diffraction (SCXRD) data (not shown) did not reveal any evident change lattice parameter nor in structure, such as the pseudo-Tsai structural modification^23^ present in some ternary systems nor the extra Cd at the Wyckoff position *8c* (assuming the space group is $Im\bar{3}$) which can occur in some rare earth-Cadmium binary R-Cd 1/1 ACs^7,10^. The structure of the LMPF Gd-Cd for $T_a$ > 400 $^{\circ }\hbox {C}$, is consistent with a predominantly 1/1 AC phase, but a slight difference of lattice parameter is observed upon close inspection, with the two samples at $T_a$ = 400, 420 $^{\circ }\hbox {C}$ sharing the same lattice parameter of $\sim$15.53 Å whereas the two samples with $T_a$ = 430, 470 $^{\circ }\hbox {C}$ have a slightly larger lattice parameter of $\sim$15.59 Å.

The LMPF-grown Gd-Au-Ge crystals were determined to be 1/1 ACs. The magnitudes of the obtained lattice parameters reflect the site-occupancy preference of some specific atomic positions^17^. Extra peaks related to Au and Ge phases appear in the PXRD patterns of some of the samples, which we relate to the presence of Au/Ge flux remaining on the samples after centrifugation, possibly due to the comparatively higher viscosity of the (Au, Ge) mixture, even close to the eutectic point.

**Fig. 4:**
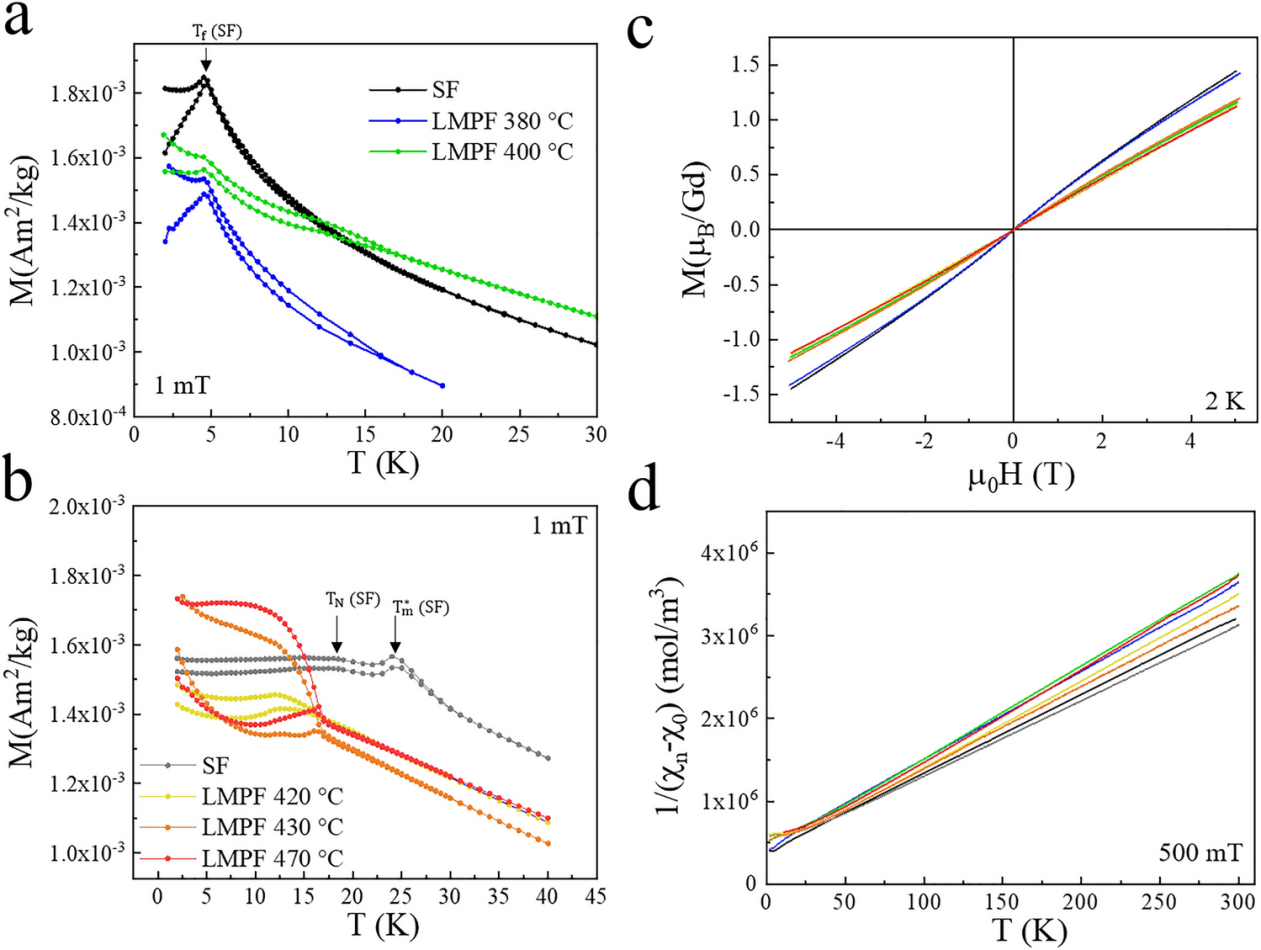
Magnetic data of SF and LMPF samples in the Gd-Cd system, with (**a**) the ZFC and FC curves of samples with low annealing temperature $T_a$ compared to the SF i-Gd-Cd QC sample and (**b**) similar plots of ZFC-FC for a high annealing temperature $T_a$ are given and compared to the GdCd_6_ 1/1 AC. (**c**) magnetization as a function of field and (**d**) inverse susceptibility plots for all samples. The color codes of (**a**,**b**) are preserved in (**c**,**d**). Note that a constant $\chi _0$ was subtracted from  χ_n_  in (**d**).

Previously, SF-grown Tsai-type Gd-Cd QCs have been found to behave like spin glasses^24^, with a likely spin glass phase transition near the freezing temperature T_f_ $\sim$ 4.7 K, see Fig. 4a. On the other hand, SF-synthesized GdCd 1/1 ACs were found to become antiferromagnetic below T_N_ = 18 K^25^, with a spurious magnetic anomaly observable in low fields near $T^*_m$ = 26 K^7,25^, as illustrated in the Supplemental Materials, see Fig. S3. The anomaly at $T^*_m$ has been identified as an indication of magnetic ordering, fully established at T_N_^25^. Note that short-ranged magnetic correlations have also been observed at even higher temperature (T $\sim$ 40 K) in heat capacity and electrical resistivity measurements^25^.

The magnetic properties for the LMPF Gd-Cd sample at 380 $^{\circ }\hbox {C}$ are very close to the QC sample obtained from SF method (freezing temperature observed at $\sim$4.7 K), see Fig. 4a, while those of the LMPF samples obtained at higher $T_a$ resembles those of the 1/1 ACs, with some qualitative differences; see Fig. 4b. Upon close inspection, the LMPF sample obtained at $T_a$ = 380 $^{\circ }\hbox {C}$ also shows excess moment below an irreversibility point of $\sim$17 K, which appears to coincide with the irreversibility of 1/1 AC samples. This may imply a small proportion of 1/1 AC phase in that sample, although not detected in PXRD. However, as expected, the LMPF sample obtained at the intermediate temperature $T_a$ = 400 $^{\circ }\hbox {C}$ shows both features of the QC and 1/1 AC phases. In the following we denotes the sample obtained at $T_a$ = 380 $^{\circ }\hbox {C}$ as LMPF QC sample, and those with $T_a$ > 400 $^{\circ }\hbox {C}$ as LMPF ACs.

The magnetic behavior of the LMPF GdCd 1/1 ACs is similar to that of the SF AC (T_N_ $\sim$ 18 K), but observed at slightly lower temperatures, akin to that observed in Y-doped^25^ and Eu-doped GdCd ACs^7^. Magnetic anomalies in the zero field-cooled (ZFC) and field-cooled (FC) curves of the LMPF samples suggest antiferromagnetic transitions at T $\sim$ 16 K (see Fig. 4b), as supported by the linear *M*(*H*) curves at low temperatures (see Fig. 4c). We note that the spurious anomaly observed at low-field^7,25^ at $T^*_m$ is absent from all LMPF samples, as seen in Fig. 4b. This anomaly has however been observed in certain Eu-doped GdCd ACs^7^.

Since there are no remarkable difference in the PXRD patterns between these samples and the SF A1/1 AC one, we speculate that the observed differences in the magnetic behavior stems from strain, surface or size effects due to the smaller grain size of LMPF samples as compared to the SF ones, as illustrated in the photographs presented in the Supplemenatry Information; the typical grain size is $\sim$ (1-5 mm)$^3$ for SF-grown systems and $\sim$ (10-50 $\mu$m)$^3$ for LMPF-grown ones^6^.

**Table 1:** Summary of the magnetic properties of SF and LMPF samples obtained from the Gd-Cd and Gd-Au-Ge systems.

Sample name	T_a_ ($^{\circ }\hbox {C}$)	a (Å)	$\theta$_CW_ (K)	p_eff_ ($\mu _B$)	$\chi _0$ ($m^3/mol$)
Gd-Au-Ge SF	–	14.7322 (13)	11.7	7.57	− 2.7 e–8
Gd_6_(Au_65_Ge_35_)_94_ LMPF	370	14.7398 (19)	11.6	5.84	− 2.6 e–8
Gd_5_(Au_70_Ge_30_)_95_ LMPF	430	14.7411 (10)	8.1	8.02	− 8.2e–8
Gd_5_(Au_60_Ge_40_)_95_ LMPF	450	14.7442 (14)	9.6	6.8	− 1.84e–7
Gd_5_(Au_60_Ge_40_)_95_ LMPF	550	14.7110 (21)	− 1.1	7.64	− 7.5e–8
GdCd_7.88_ SF	–	7.972 (1) ($a_{6D}$)	− 39.7	8.2	− 8.0e–8
Gd_5_Cd_100_ LMPF*	380	7.972 (1) ($a_{6D}$)	− 41.4	7.88	− 1.5e–8
Gd_5_Cd_100_ LMPF	400	15.5199 (7)	− 35.1	7.54	− 1.7e–8
Gd_5_Cd_100_ LMPF	420	15.5237 (7)	− 34.6	7.81	− 1.5e–8
Gd_5_Cd_100_ LMPF	430	15.5952 (12)	− 37.4	7.87	− 7e–8
Gd_5_Cd_100_ LMPF	470	15.5936 (10)	− 37.5	7.81	− 3.3e–8
GdCd_6_ SF	–	15.5210 (15)	− 31.5	8.14	− 6e–9

$T_{a}$ refers to the annealing temperature. In the case of both GdCd_7.88_ and the Gd_5_Cd_100_ LMPF sample with $T_a$ = 380 K, the QC hyperspace lattice parameter $a_{6D}$ is given instead of the lattice parameter^2^ ($a_{1/1}=a_\textrm{6D}\sqrt{\frac{2}{2+\tau }}(1+\tau )$ where $\tau$ is the golden ratio^5^). We have considered uncertainties of ± 0.5 K on $\theta$_CW_ and ± 0.05 $\mu _B$ on p_eff_. $^{*}$ p_eff_ calculated assuming the molecular weight of GdCd_7.88_.

For all samples, Curie-Weiss fits were performed assuming the formula $$\chi (T) = \frac{C}{T - \theta _{CW}} + \chi _0$$, where $$\chi _0$$ represents a temperature-independent susceptibility, see Fig. 4d. The parameters were obtained from the fits of the data between in the linear region T = 50 K and T= 250 K from the inverse susceptibility, and listed in Table 1. Examples of fits are shown in the supplementary information (Fig. S4). The obtained effective moments are close to the theoretically expected ones ($$\mathrm p_{eff}^{theo} =$$$$g\sqrt{J(J+1)} \mu _B$$ $$\sim$$ 7.94 $$\mu _B$$ considering a 4$$f^7$$ configuration for Gd), and as observed earlier, the Curie-Weiss temperatures are slightly larger for the QCs than for the ACs^25^. The *M(H)* curves of the SF and LMPF crystals are either linear or slightly non-linear (see Fig. 4c). Similar to previous reports on Y- and Eu-doped samples^7,25^, we observe a linear behavior in the *M(H)* curves of all 1/1 ACs whereas a slightly “S-shaped” curve is characteristic of the QCs and their glassy behavior.

**Fig. 5:**
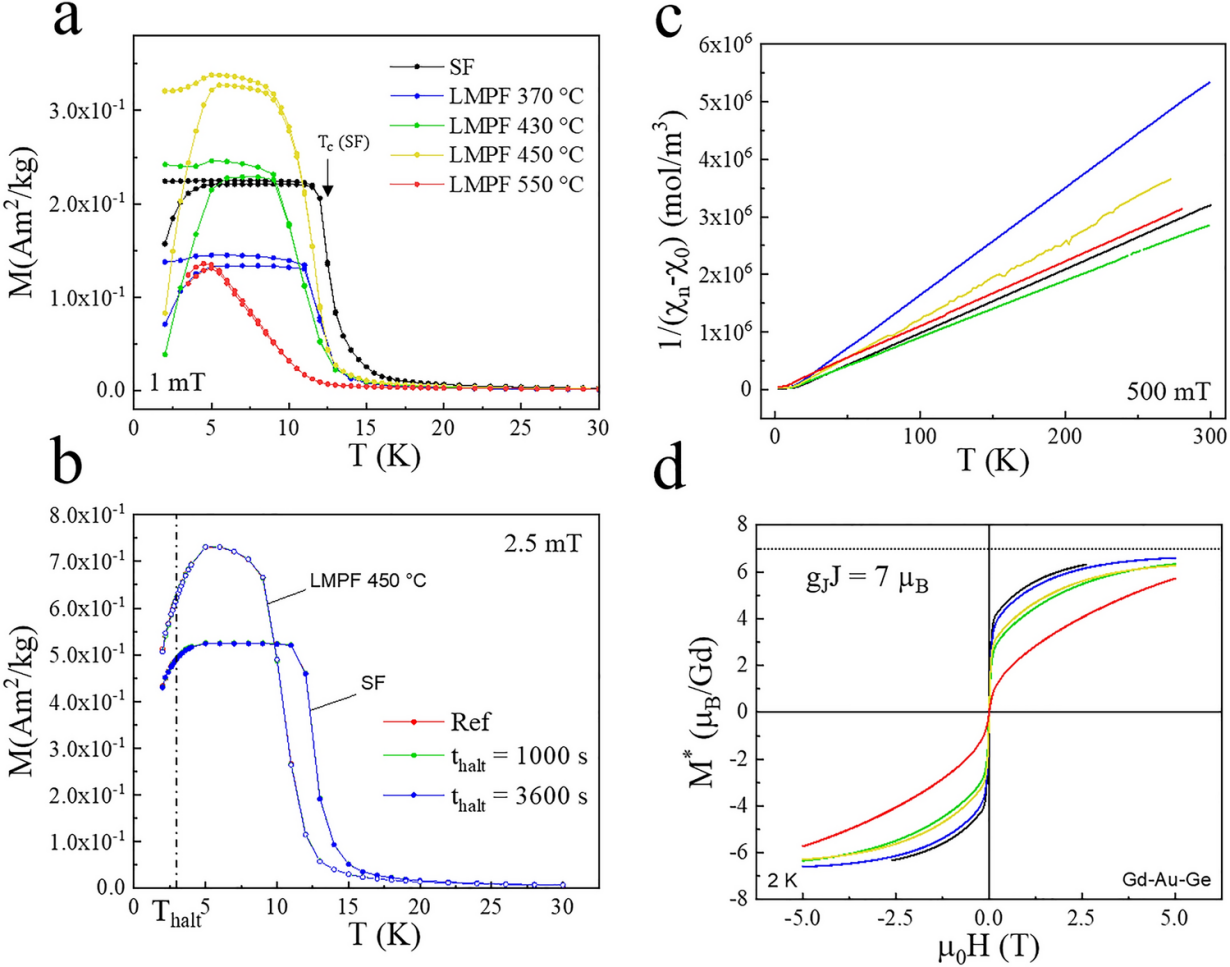
Magnetic data of SF and LMPF samples in the Gd-Au-Ge system, with (**a**) the ZFC and FC curves of all LMPF samples synthesized compared to the SF Gd-Au-Ge sample. (**b**) Memory effect attempts for different halt times $t_{halt}$ performed at the temperature $T_{halt}$ = 3 K in both the SF Gd-Au-Ge sample and the LMPF sample annealed at $T_a$ = 450 $^{\circ }\hbox {C}$. (**c**) The inverse susceptibility plots and (**d**) renormalized magnetization as a function of field plots for all samples, i.e. $M^* = M\times (7.94/p_{eff})^2$ with $p_{eff}$ extracted from linear fits of the inverse susceptibility. The color codes of (**a**) are preserved in (**c**,**d**).

The SF-grown crystals of the Gd-Au-Ge 1/1 AC display a ferromagnetic state below T_c_ = 15 K, which seems to be rearranged at lower temperatures^9^, see Fig. 5a. In the ternary Gd-Au-Ge 1/1 AC^9^, there exists no competing phase in the lower temperature range, and the magnetic properties shall not be affected by a mixture of different phases if $T_a$ is chosen in the low temperature region. We have investigated different precursor compositions Au_*z*_Ge_100-*z*_, yielding LMPF samples with different Au concentration *z* in the range of z = 60-70, close to the eutectic point. Their magnetic behavior is consistent with a ferromagnetic transition at a temperature similar to that of the SF AC, except for the AC obtained using a high annealing temperature T_a_ = 550 $^{\circ }\hbox {C}$; possibly reflecting another type of magnetic order. The magnetic behavior of the AC obtained at T_a_ = 550$^{\circ }\hbox {C}$ is reminiscent to that reported for an hexagonal Gd-Au-Ge phase^26^ with T_c_ = 10-13 K. Note that the magnetic susceptibility of the SF AC and LMPF ACs with T_a_ < 550$^{\circ }\hbox {C}$ appears to be limited by demagnetizing effects^18,27^ below T_c_. A sharp drop is observed at lower temperatures, which was attributed to a reentrant spin glass behavior^9^. A method to assess whether a material displays a spin glass behavior is to perform memory experiments^28,29^, where one compares temperature dependent zero field-cooled magnetization curves *M(T)* recorded with and without a halt during the cooling. If the system displays glassy dynamics at the halt temperature, its magnetic configuration will slowly rearrange itself (slow drift to equilibrium). The aging occurring during the halt will be kept in memory upon resuming the cooling, inside a temperature region around the halt temperature; outside that range the system appears reinitialized (rejuvenated) and displays the same response as in a reference measurement recorded without halt^28^. The aging, memory and rejuvenation phenomena will thus imprint a “dip” in the *M(T)* curves recorded including a halt^28^. These features are characteristic of systems undergoing magnetic phase transitions from paramagnetic at high temperatures to spin glass at low temperatures (spin glasses), or from paramagnetic to an intermediate ordered yet frustrated (e.g. ferro-) magnetic phase at lower temperatures, turning into a spin glass at even low temperatures (reentrant spin glasses^30^). Memory experiments have successfully been carried out in quasicrystals^24,31^ and approximants^20^. Memory experiments were thus performed on the SF and one of the LMPF samples to investigate the possible glassiness of the Gd-Au-Ge approximants at low temperatures, i.e. a potential reentrant spin glass behavior. As shown in Fig. 5b, we did not observe any difference between M(T) curves recorded with and without halt during the cooling, suggesting the lack of glassy behavior in these ACs (SF or LMPF). The observed decrease of the magnetization at low temperatures in Gd-Au-Ge shall may thus instead reflect a transition or rearrangement from the ferromagnetic state to a more complex and more anisotropic magnetic state^16^.

The magnetization curves as a function of field for all the Gd-Au-Ge crystals, and inverse magnetic susceptibility from which the effective moment $p_{eff}$ and Curie-Weiss temperature $\theta$_CW_ were estimated are shown in Fig. 5c (see Table 1 for values). We speculate that the effective moment of the ACs shall be close to the theoretically expected one ($\sim$ 7.94 $\mu _B$ as in the Gd-Cd system), as observed in most of the QCs and ACs^3,32^. The difference in slopes (proportional to the inverse of the Curie constants) observed in Fig. 5c hence stems from the Gd|(Au+Ge) ratio, which is not the nominal one. The effective Gd concentration may hence be inferred from the values of the Curie constants (C $\propto$ $n_{Gd} p_{eff}^{2}$; $n_{Gd}$ being the Gd concentration), and used to renormalize the M(H) curves, as shown in Fig. 5d (See the Supplemental Material for the M(H) curves plotted considering the nominal Gd compositions; see Fig. S5).

Overall, in the Gd-Au-Ge 1/1 AC system, as opposed to the Gd-Cd 1/1 AC, the magnetic behavior is preserved in LMPF samples. This may be due to Gd-Au-Ge samples already having disorder (chemical mixing) present in their structure, whereas the Gd-Cd 1/1 ACs do not. Therefore, quenched disorder may affect more dramatically the latter. The absence of a stable QC makes unlikely the existence of a competing phase. A summary of the magnetic properties of all samples is presented in Table 1.

Different concentrations *z* of the precursor Au_*z*_Ge_*100-z*_ allows to shift downward the centrifuging temperature of the samples synthesized, with the lowest point being at the eutectic point at Au_72_Ge_28_, and a melting point of 361 $^{\circ }\hbox {C}$, slightly above the melting point of Cd. Remaining flux can be an issue in the ternary samples, as opposed to the binary systems, but increasing too much the annealing temperature to $T_a$ = 550 $^{\circ }\hbox {C}$ appeared to affect the magnetic behavior of the LMPF sample. We suggest the reason might be a different competing phase appearing at this temperature^26^.

## Conclusions

In conclusion, we have synthesized and characterized QC and 1/1 AC samples in the binary Gd-Cd and ternary Gd-Au-Ge systems, using both a self flux and low-melt peritectic method (quenching and annealing) for comparison. The overall magnetic properties of the LMPF-grown crystals were found to be relatively similar to their SF-grown counterpart, especially in the Gd-Au-Ge case.

The magnetic order sets in at a slightly lower temperature in the Gd-Cd system, and the magnetic susceptibility has a different irreversibility with temperature. The LMPF-grown Gd-Cd samples present identical PXRD and SCXRD patterns as the SF-grown ones, suggesting annealing-resistant disorder or grain size-related effects. In the case of Gd-Au-Ge compounds, the magnetic properties of the 1/1 ACs synthesized using LMPF were preserved in all crystals except for the one obtained using the highest annealing temperature $T_a$ = 550 $^{\circ }\hbox {C}$. The presence of an intrinsic chemical disorder in the ternary system may explain why the magnetic properties of the LMPF crystals are better preserved in comparison to the binary Gd-Cd 1/1 ACs ones.

Owing to the very large diversity of ternary systems in terms of chemical elements, and the infinite number of chemical ratios achievable, we believe that methods such as LMPF, enabling fast synthesis for the exploratory research of promising materials, can facilitate the discovery of new QCs and ACs with interesting physical properties.

## Methods

All samples were obtained from highly pure (>99.99%) Gd, Cd, Au and Ge reactant elements commercially obtained from STREM and CHEMPUR companies, in chip or powder form. Arc-melted ingots of (Au, Ge) at the eutectic point of the phase diagram were preliminarily made for the synthesis of ternary Gd-Au-Ge samples. In the case of self-flux samples, after weighing the nominal amounts in an argon filled glove box (O_2_ < 0.1 ppm volume), the reactants were then transferred to a cylindrical alumina crucible, which was then sealed inside a stainless steel tube. Both ends of the stainless steel tube were welded in an argon filled atmosphere in order to keep the samples in an inert atmosphere. First, the reactants were heated to 700 $^{\circ }\hbox {C}$ and left for a few hours at that temperature to ensure homogenous melts, then slowly cooled to their final temperature and left to anneal before centrifuging the single crystal grains. The samples obtained from low-flux peritectic melt method were heated in a similar manner to obtain homogeneous melts, but first directly quenched in liquid N_2_, then reheated to their final temperature and left to anneal for 100 hours before centrifuging the samples from the melt. Optical microscope pictures of SF- and LMPF-grown samples are shown in Fig. S1. The characterization of the samples was done using powder X-ray diffraction (PXRD) using a Bruker D8 Powder diffractometer. The lattice parameters were determined and indexed from the PXRD patterns. PXRD patterns were indexed and refined using CheckCell^33^. The magnetic data was acquired using a superconducting quantum interference device (SQUID) from Quantum Design Inc., model MPMS XL.

## Supplementary Information

Supplementary Information.

## Data Availability

The datasets used and/or analyzed during the current study available from the corresponding author on reasonable request.
