# Supplementary material for: Magnetic properties of quenched binary and ternary quasicrystal approximants
Source: Sci Rep. 2024 Dec 17;14:30538. doi: 10.1038/s41598-024-81987-7 (PMC11652634; doi:10.1038/s41598-024-81987-7)
Supplement: Supplementary file 1 — Supplementary Information. [file 41598_2024_81987_MOESM1_ESM.pdf]

Supplementary Information

Magnetic properties of quenched binary and ternary quasicrystal approximants

Fernand Denoel[1,*], Takayuki Shiino[1], Yu-Chin Huang[2], Girma Hailu Gebresenbut[2], Cesar Pay Gómez[2], and Roland Mathieu[1*]

[1]Department of Materials Science and Engineering, Uppsala University, Box 35, 751 03, Uppsala, Sweden
[2]Department of Chemistry - Ångström laboratory, Uppsala University, 751 21 Uppsala, Sweden

*fernand.denoel@angstrom.uu.se ; roland.mathieu@angstrom.uu.se

This Supplemental Material includes additional pictures, PXRD patterns and magnetic properties data for the Gd-Cd and Gd-Au-Ge crystals synthesized with self-flux and low-melt peritectic formation methods.

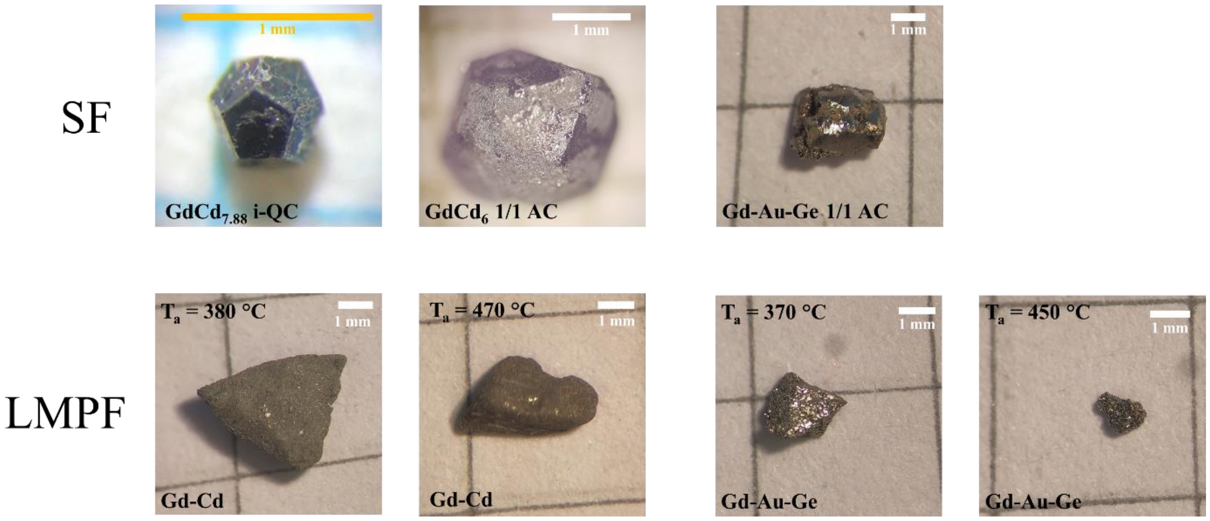

Figure S1: Microscope pictures of (top) SF- and (bottom) LMPF-grown samples in the Gd-Cd and Gd-Au-Ge systems.

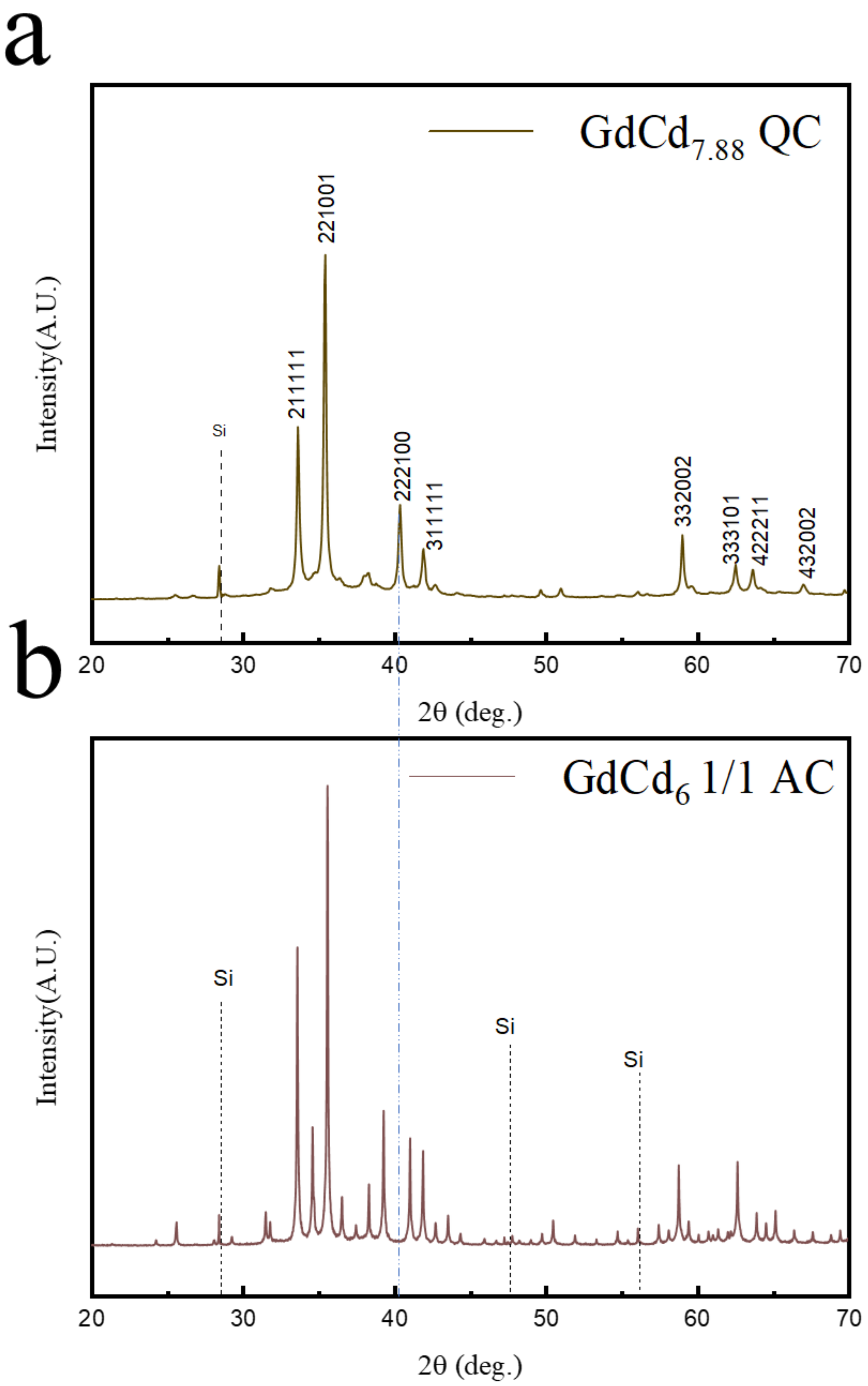

Figure S2: PXRD patterns of SF-grown (a) the binary $GdCd_{7.88}$ QC and (b) the binary $GdCd_6$ 1/1 AC. The dashed line spanning both panels denotes the same QC-only reflection as the one highlighted the main text.

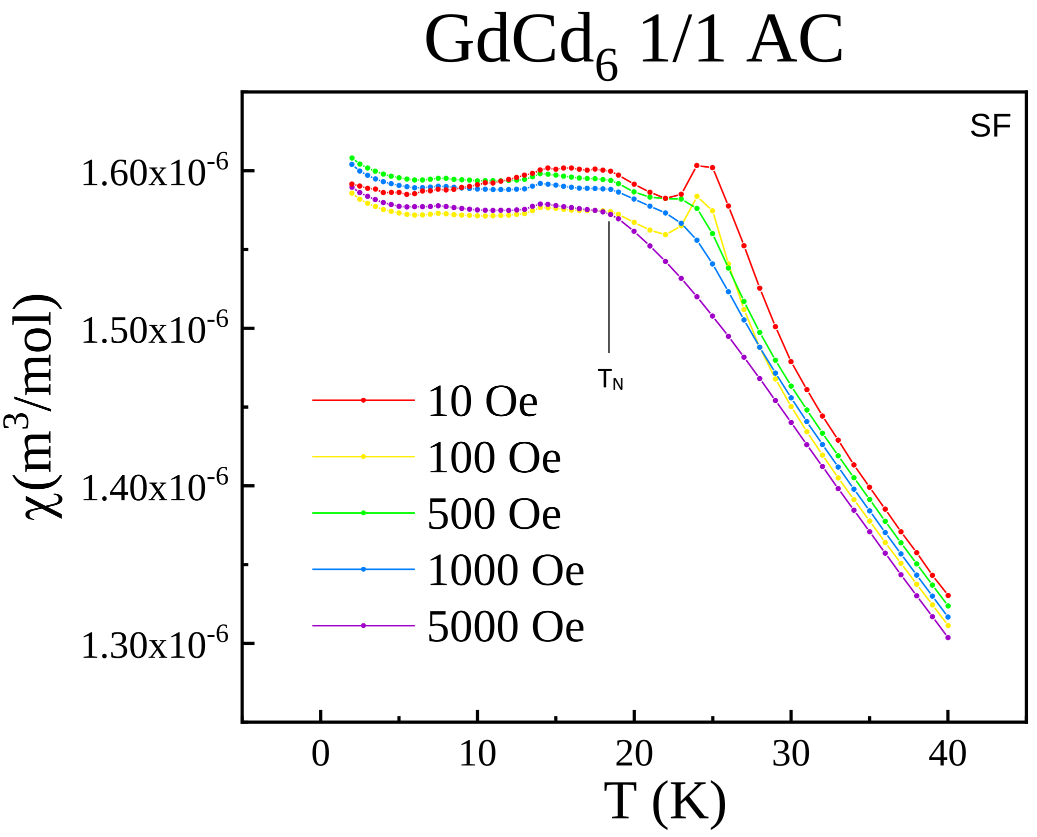

Figure S3: Observation of the spurious magnetic ordering above the Néel temperature in a SF sample of the $GdCd_6$ 1/1 AC. Under high field, this effect is suppressed. Data from Ref. [1]

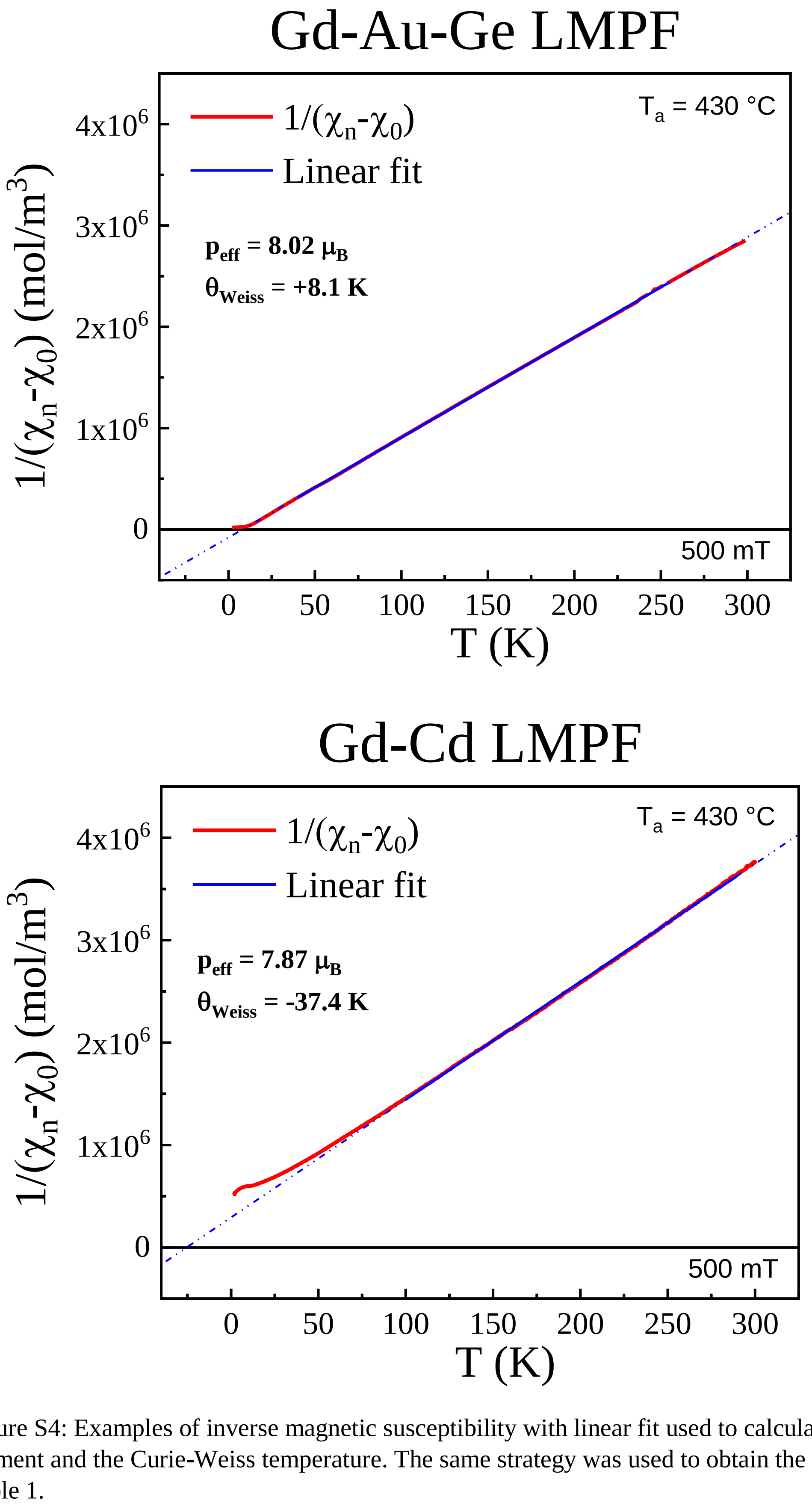

Figure S4: Examples of inverse magnetic susceptibility with linear fit used to calculate the effective moment and the Curie-Weiss temperature. The same strategy was used to obtain the values found in Table 1.

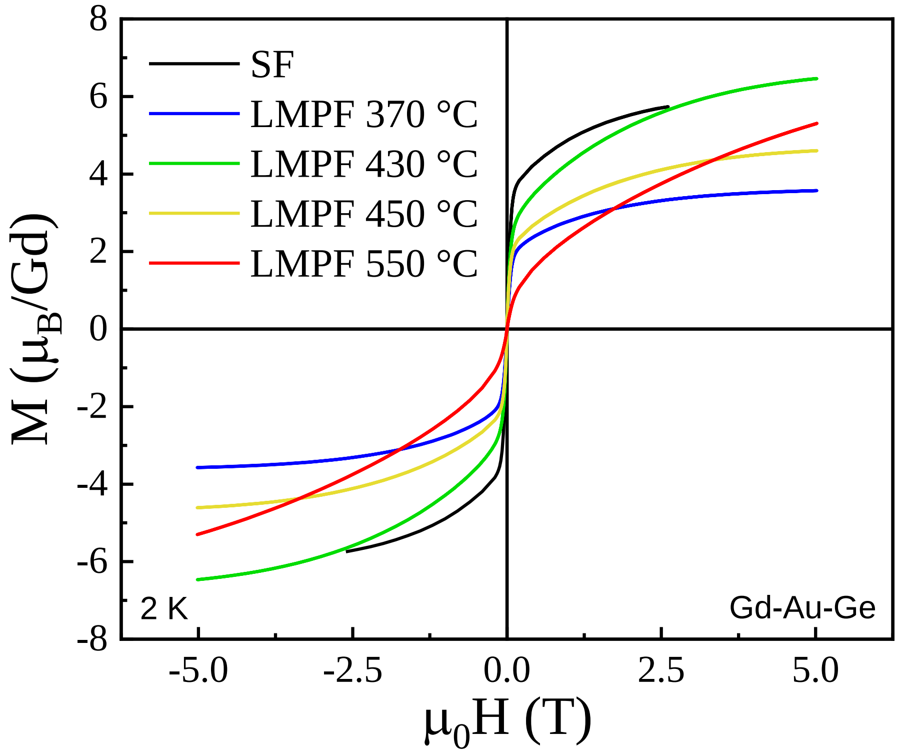

Figure S5: Magnetization per Gd element as a function of field applied in the Gd-Au-Ge samples. No normalization procedure was performed.

[1] T. Shiino, F. Denoel, G. H. Gebresenbut, D. C. Joshi, Y. C. Huang, C. P. Gómez, U. Häussermann, A. Rydh, and R. Mathieu, *Singular Magnetic Dilution Behavior in a Quasicrystal Approximant*, Phys. Rev. B **104**, 224411 (2021).
